# A case of pleural Mycobacterium tuberculosis infection with reversion of Quantiferon Gold Plus results from positive to negative

**DOI:** 10.1099/acmi.0.000737.v3

**Published:** 2024-09-05

**Authors:** N. Goire, M.S. Suchard, A. Barling, R. Fernando, L. Dreyer, A.A. Mahony

**Affiliations:** 1 Microbiology Department, Australian Clinical Laboratories, Clayton, Victoria, Australia; 2 University of Wollongong Faculty of Health and Behavioural Sciences: University of Wollongong, Wollongong, New South Wales, Australia; 3 Chemical Pathology Department, School of Pathology, University of the Witwatersrand, Johannesburg, South Africa; 4 Thoracic Surgery Department, Bendigo Hospital, Bendigo Health, Bendigo, Victoria, Australia; 5 Histopathology Department, Australian Clinical Laboratories, Bendigo, Victoria, Australia; 6 Infectious Diseases Unit, Bendigo Health, Bendigo, Victoria, Australia; 7 Infectious Diseases Department, Austin Hospital, Heidelberg, Victoria, Australia

**Keywords:** IGRA, quantiferon gold, *Mycobacterium tuberculosis*, PCR, pleural TB, reversion

## Abstract

**Introduction.***Mycobacterium tuberculosis* (MTB) infections continue to have a high mortality and morbidity burden globally. Interferon-gamma release assays such as Quantiferon Gold Plus (QFG-Plus) aid in diagnosis of latent TB but diagnosis of pleural TB remains challenging. We present a case of active pleural MTB infection with reversion from positive to negative of IGRA result as well as negative Xpert MTB/RIF Ultra PCR result from tissues obtained from pleural biopsy.

**Case summary.** A 52-year-old otherwise healthy male presented in August 2022 with a 2 week history of pleuritic chest pain associated with modest elevation in inflammatory markers. The patient had had a positive QFG-Plus result in 2018, however QFG-Plus during this admission was negative. Computed-tomography pulmonary angiogram and needle thoracocentesis showed an exudative left pleural effusion with predominant lymphocytes. The patient’s symptoms failed to resolve with empiric antimicrobial therapy for community-acquired pneumonia. Broncho-alveolar lavage as well as biopsies of pleural tissues via video-assisted thoracoscopic surgery from the left lower lobe yielded negative results on routine microbiological culture as well as Xpert Ultra PCR. Growth of acid-fast bacilli was noted from mycobacterial cultures of pleural tissues which was identified as MTB.

**Conclusion.** Despite significant technological advances, microbiological diagnosis of MTB infections remains challenging. We document QFG-Plus reversion during development from latent to active pleural TB. Decline in the ability of CD4^+^ and CD8^+^ T cells to produce interferon gamma in response to TB antigens (ESAT-6 and CFP-10) was likely associated with loss of host control of latent MTB. This case serves as a reminder that despite exhaustive testing with state-of-art diagnostic platforms, MTB infections can still elude discovery.

## Data Summary

No data was generated during this research, or is required for the work to be reproduced.

## Introduction

With a vast footprint, multitudes of manifestations, and worsening antimicrobial resistance, *Mycobacterium tuberculosis* (MTB) retains its infamous distinction as a scourge of the under-privileged. Modern day geo-political upheavals, developments in travel and transport and a long latency period mean that the mycobacterium has been reported from areas well outside of where it is endemic, and from the farthest reaches of the world. Per the World Health Organisation (WHO), MTB worldwide incidence for the year 2021 was about 134 cases per one million persons [1]. Although Australia falls outside of global hotspots or high-incidence zones for MTB, an estimated 1500 people are diagnosed with tuberculosis (TB) each year, with Victoria accounting for about 450 to 500 of these cases [2]. Whilst the majority of TB diagnosed in Australia is of pulmonary origin, extra-pulmonary TB accounts for approximately 40% of notifications [3]. Pleural TB particularly presents a special diagnostic challenge due to its atypical course and the low pathogen burden in pleural spaces [4,6].

Laboratory diagnosis, and the expertise required to diagnose MTB infections, are therefore critical to early management. Understanding both the advantages and pitfalls of three of the most widely used diagnostic platforms – namely mycobacterial culture, Interferon Gamma Release Assays (IGRA) and nucleic acid amplification tests such as Xpert MTB/RIF Ultra (Xpert Ultra) (Cepheid, Sunnyvale, CA, USA) is crucial in navigating the complexities posed by pleural MTB infection.

## Case report

A 52-year-old male presented to a regional hospital in Australia in July 2022 with a 2 week history of pleuritic, left-sided chest pain, febrile episodes and dyspnoea. Inflammatory markers were moderately if at all elevated: C-reactive protein 61 mg l^−1^ and white cell count was 5.5×10 ^9^ per litre. Investigations for a cardiogenic cause of chest pain were unremarkable. d-Dimer was elevated at 9670 ng l^−1^ (normal range <500 ng l^−1^) and radiological investigations showed a left sided pleural effusion without pulmonary embolism. Urine antigen tests for legionella and pneumococcus were negative.

The patient did not harbour any significant risk factors for MTB other than having lived in Spain for much of his life before migrating to Australia in 2010, and working in healthcare but there was no known history of TB contacts. An interferon gamma release assay, the Quantiferon Gold Plus (Qiagen Inc, Australia) (QFG-Plus) performed in July 2018 as a part of pre-employment screening had provided a positive value (TB1-nil=0.66, TB2-nil 0.84, cut-off 0.35); latent TB infection treatment was inadvertently not offered. Repeat QFG-Plus in August 2022 was negative (TB1-nil=0.04, TB2-nil=0.16) as was HIV serology.

Pleural fluid obtained via thoracocentesis was exudative (LDH ratio of effusion to serum=4.9), with a white cell count of 5400×10^6^ per litre, predominantly (50%) lymphocytes, associated with an erythrocyte count of 63 000×10^6^ per litre. Gram-stain showed no bacteria and there was no microbial growth on routine bacterial culture. Direct smear for acid-fast bacilli as well as Xpert Ultra were negative from the pleural fluid. Cytology excluded malignancy.

**Fig. 1.**
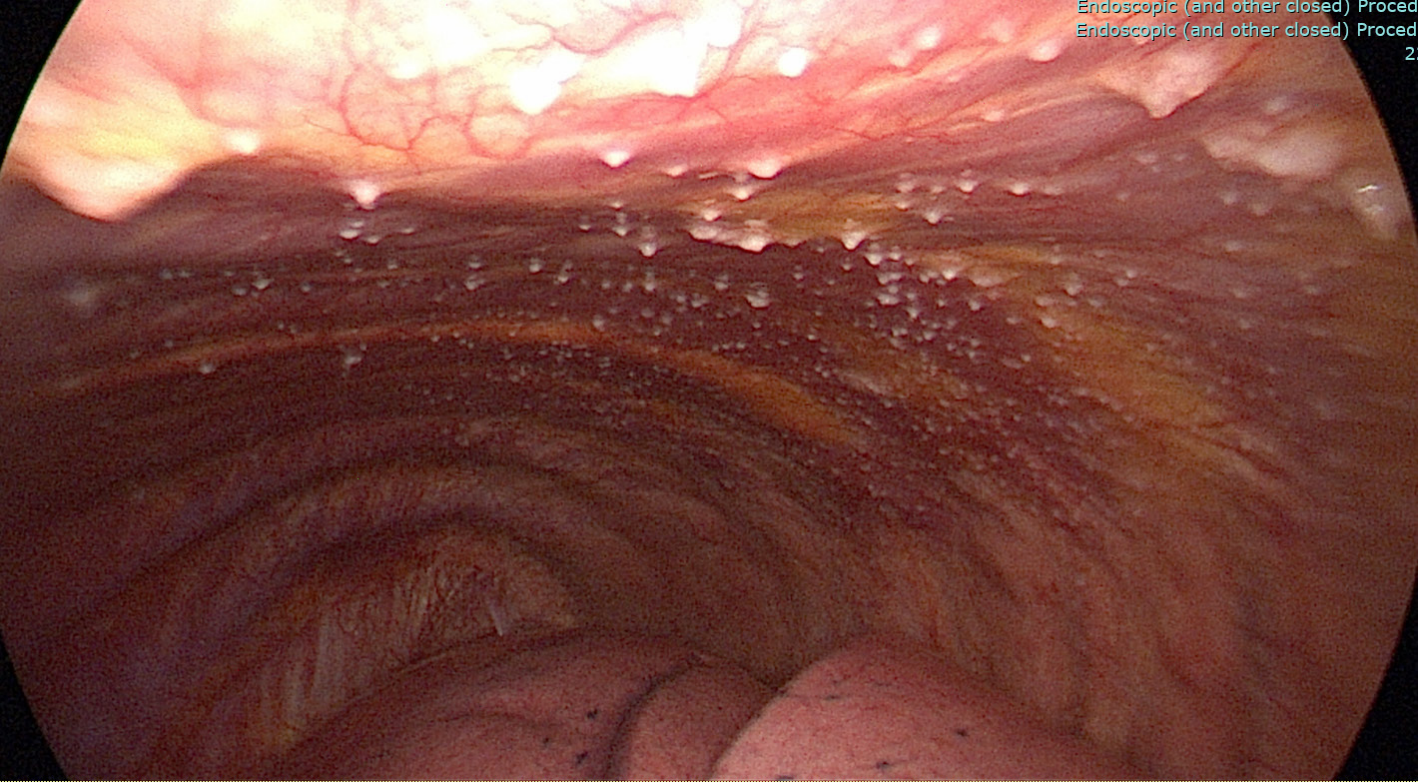
Thorascoscopic images of parietal pleura. Image showing multiple acneiform lesions over the parietal pleura.

The patient was commenced on empiric ceftriaxone and azithromycin in the Emergency Department, then discharged home on amoxicillin plus doxycycline for outpatient follow-up. Symptoms were unabated, and repeat chest x-ray showed enlargement of the pleural effusion. Bronchoscopy was unremarkable macroscopically, whereas video-assisted thoracoscopic decortication showed multiple small, acneiform lesions over the left lower lobe parietal pleura (Fig. 1). These lesions were biopsied and a Broncho-Alveolar Lavage (BAL) was collected. Both BAL and pleural tissues were negative by Xpert Ultra as well as direct smear for acid-fast bacilli. Routine microbial cultures did not yield any growth after extended incubation. Based on the parietal pleura macroscopic findings, the patient was commenced on standard first-line anti-TB treatment.

**Fig. 2.**
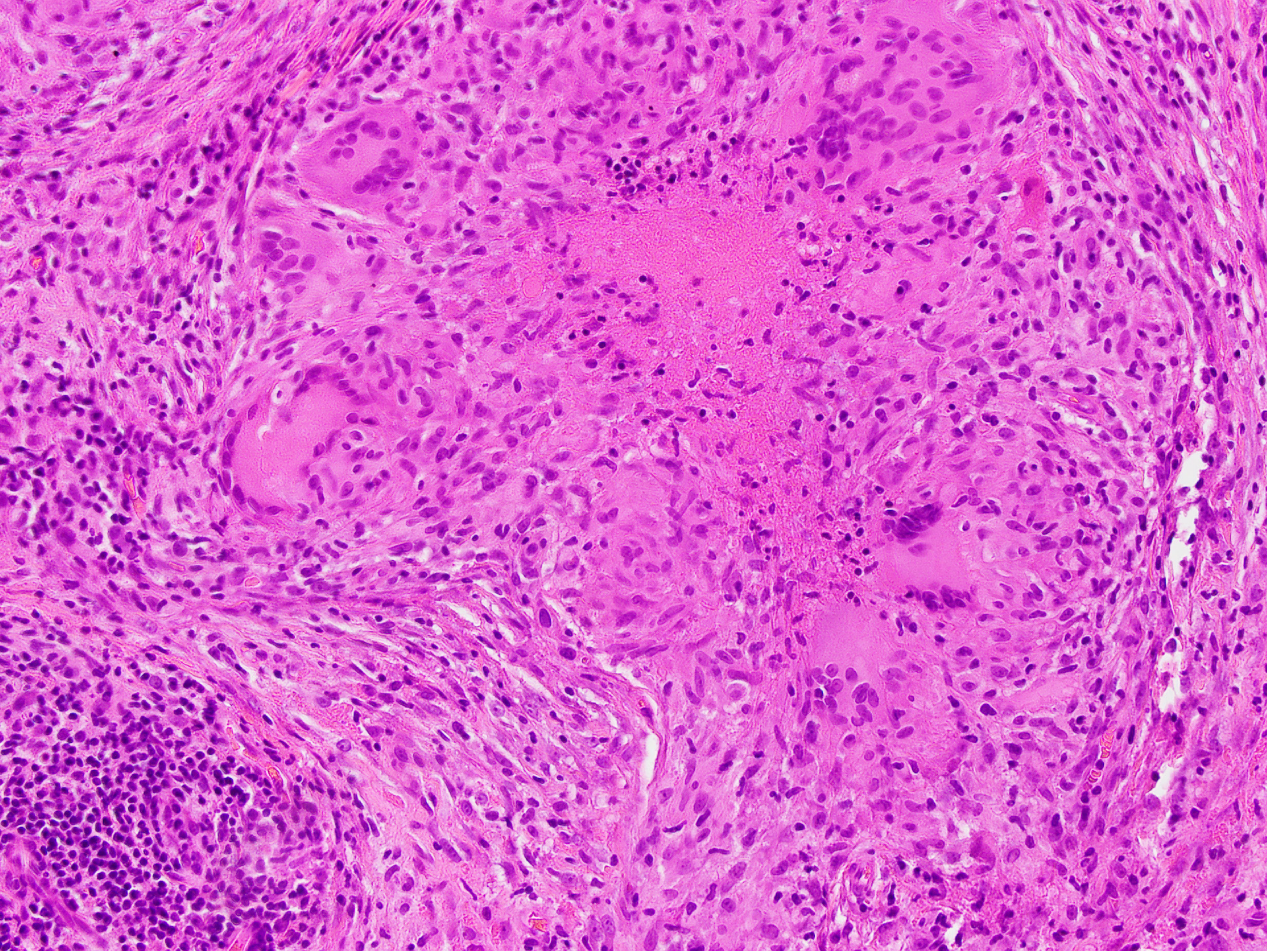
Left lower lobe parietal pleura histopathology slide. Haematoxylin and eosin stain of pleural tissue biopsy showing multiple histiocytic granulomas with giant cells and central necrosis.

**Fig. 3.**
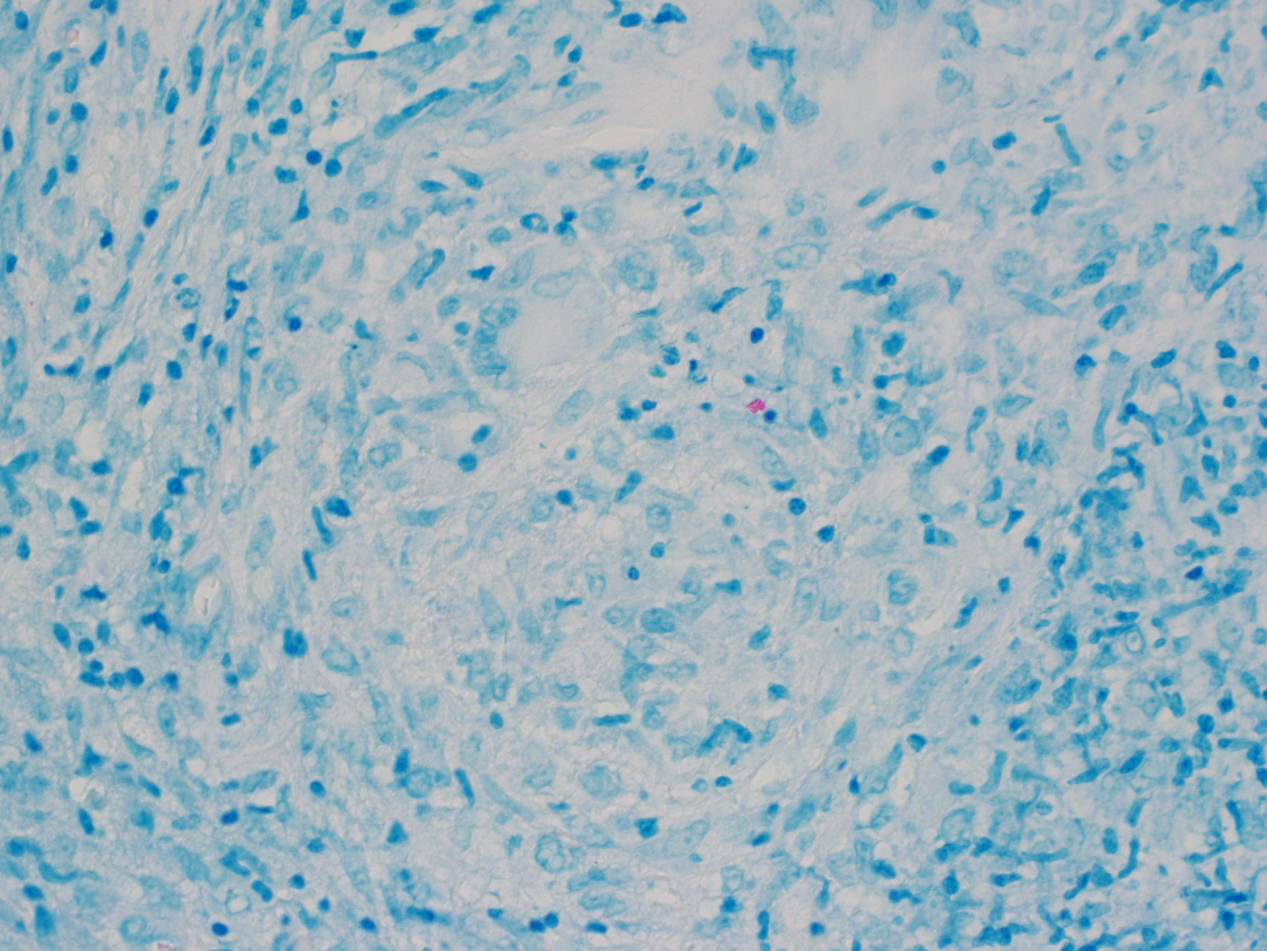
Left lower lobe parietal pleura biopsy. Ziehl-Neelsen stain of pleural tissue biopsy showing a solitary, positive cluster.

Histopathological analysis of pleural tissue showed granulomatous inflammation, with lesions composed of epithelioid histiocytes, Langhans-type giant cells surrounded by cuff of lymphocytes arranged within fibrous stroma. Granulomas also exhibited central necrosis (Fig. 2). A further Ziehl-Neelsen stain of the histological sections showed occasional acid-fast bacilli, supporting the diagnosis of pleural MTB disease (Fig. 3). Mycobacterial culture of the pleural tissues flagged positive after 7 days’ incubation in liquid agar medium (MGIT-960); Ziehl-Neelsen stain of the growth medium showed acid-fast bacilli. Xpert Ultra was positive for MTB and negative for the *rpo*B gene mutation. Anti-mycobacterial susceptibility tests performed on this isolate showed sensitivity to rifampicin, isoniazid, ethambutol and pyrazinamide. The patient continued standard treatment for 6 months with resolution of symptoms and pleural effusion, and remains well at twelve months’ follow-up. Follow up QFG-Plus, performed a year post-presentation, remains negative.

## Discussion

Per WHO definitions, pleural TB is considered as extra pulmonary rather than pulmonary TB [7]. Pleural biopsy rather than pleural fluid is the specimen of choice for diagnosis. For pleural fluid, Xpert Ultra has sensitivity under 50%, but high sensitivity approaching 100% for pleural biopsy [8]. The WHO has recommended the Xpert Ultra assay for diagnosis of TB from extra-pulmonary samples, and on pleural fluid or biopsy as well as for children and HIV-infected patients [9,10].

QFG-Plus is an assay investigating the production of interferon gamma (IFN*γ*) in response to *in vitro* stimulation with the MTB antigens. Results are first compared by subtracting the value obtained from an unstimulated tube (nil tube). Different lengths of the stimulating peptide antigens in tubes one and two are intended to stimulate both CD4^+^ (TB1 tube) and CD8^+^ T cells (TB2 tube). The QFG-Plus is interpreted as positive when the IFN-γ concentration of the TB antigen tube (TB1 or TB2) minus the unstimulated tube (Nil tube) is ≥0.35 IU ml^−1^ and ≥25% of the nil value [11]. Earlier versions of the assay, QuantiFERON-TB Gold or QuantiFERON-TB Gold In-tube (QFG-GIT) lacked the TB2 tube [12,13].

IGRAs perform better as tests of TB exposure rather than diagnostic tests for active TB disease. In active disease, results vary greatly. Studies as early as 2007 indicated that sensitivity for diagnosis of active TB diseases was suboptimal, with negative IGRA results more common amongst cases of extra-pulmonary TB than pulmonary TB [6]. The QFG-Plus assay is therefore not advised for diagnosis of active TB disease [12]. For example, Bartu *et al*. reported positive QFG results in only 73–81% of culture-confirmed TB cases and slightly higher sensitivity than the older version QFG-GIT for predicting active TB (82.5% vs. 77.8%) [14,17]. Site of TB disease may be a factor influencing false negative IGRA results. In patients with proven TB, negative QFG-GIT results were found in 28.6% of pleural, 8.3% of lymph node, 8.3% of skeletal and 5.8% of gastrointestinal TB cases [18].

In our case, the documented positive QFG-Plus in 2018, followed by a negative result at diagnosis, is of interest. Some institutions would consider the 2018 TB1 value of 0.66 as low positive, with values above 0.7 as high positive. Nonetheless the high positive TB2 value was conclusively positive and diagnostic of latent TB, which indeed proved to be true by later development of active disease. In 2022, the negative QFG-Plus result suggests that lack of functionality in CD4^+^ and CD8^+^ T cells to TB antigens was associated with the development of active TB disease. This concept is sometimes termed ‘T cell anergy’ and is well described as a caveat when interpreting any immune response assay to TB, including the older tuberculin skin test. Supportive flow cytometric work has suggested that active TB is sometimes associated with lower, rather than higher, T or B cell responses to TB antigens. For example, Chiacchio *et al*. showed that CD8^+^ T cells responding to TB antigens were almost absent at TB diagnosis, returning to higher levels after treatment [19]. Buldeo *et al*. found that individuals with active TB disease produced significantly less IFN*γ* in CD4^+^ T cells from induced sputa in response to Purified Protein Derivatives (PPD) stimulation than controls and suggested that progression to active TB disease may be associated with loss of IFN*γ* secretion from helper T cells at the site of primary infection [20]. Despite return to health, the QFG-Plus in our patient remained negative 1 year later. The issue of negative IGRA results following positive IGRAs has been termed ‘reversion’. In a prospective study of healthy individuals, active TB disease incidence was higher in QFG-GIT reverters (1.47 per 100 person years) than in those who were either persistently QFG-GIT negative (0.18 cases per 100 person years), QFG-GIT positive (1.05 per 100 person years) results, or than converters from a negative to a positive QFG-GIT result (1.39 per 100 person years) [21]. Our case fits the interpretation that conversion from positive to negative IGRA can be a warning sign for development of active disease. Meta-analysis has however shown that rate of QFG-GIT reversion also occurs in approximately 25% of asymptomatic individuals with an initial positive QFG-GIT result [22]. A better understanding of longitudinal IGRA fluctuations should allow improved interpretation of results in patients with suspected disease. In our case the baseline result prior to development of symptoms was a helpful tool.

IGRAs and rapid-diagnostic platforms such Xpert Ultra have become the mainstay of laboratory diagnosis of MTB infections in resource-rich settings. In a population such as Australia where prevalence of MTB infections is relatively low, the positive predictive values of these tests are consequently low, and they should exhibit higher negative predictive values. Cases of negative IGRA and PCR results with positive culture have previously been reported in the literature, particularly from high prevalence settings. The gold standard test remains mycobacterial culture. In addition, higher mortality is noted in MTB patients where the IGRA is negative, owing mostly to delayed therapeutic interventions [23]. Commencement of anti-mycobacterial therapy, in scenarios where thoracoscopic appearance of the pleura is suggestive of MTB disease, and in absence of any other positive MTB-specific tests is therefore the correct course of action.

Our case represented an interesting scenario where the extra-pulmonary (parietal pleura) manifestation of MTB was not detected by PCR on biopsies of the lesions and yet had a positive histopathology finding and eventually a positive mycobacterial culture. The Xpert Ultra PCR, as expected, was positive on cultured isolate. Low mycobacterial load may have led to negative Xpert Ultra PCR result on the clinical specimens, a phenomenon well described in pleural TB [5]. Alternative nucleic acid formats such as loop-mediated isothermal amplification, TrueNAT TB and cell-free DNA have been considered to address the challenges of pleural TB but were not available in our setting [5,24]. Other biomarkers in pleural fluid may also be informative, such as unstimulated interferon gamma, adenosine deaminase or adenosine deaminase-2, with unstimulated interferon gamma performing the best of the three markers [25,26]. Stool has also been used in certain patient populations for diagnosis of extrapulmonary TB [27]. The Xpert Ultra is a fully nested, cartridge-based real-time PCR assay targeting IS6110, IS1081 and the rpoB gene for simultaneous detection of rifampicin resistance. It has increased sensitivity than the semi-nested Xpert MTB/RIF assay [28]. It is in this setting that the WHO recommends, Xpert Ultra on paediatric, smear-negative/culture-positive specimens and cerebro-spinal fluids [10].

A limitation of our study is that no residual 2018 serum was available for repeat testing in 2022. Factors that can affect the performance of IGRAs include pre-analytical factors such as the need to collect the mitogen control tube last to avoid potential carry over of mitogen to other tubes, requirement for sufficient volume per tube and adequate mixing of the tubes at the bedside [11]. Following initial positive TB IGRA result, the patient was lost to follow up, and later presented with active extra-pulmonary TB. It wasn’t until the bronchoscopy images strongly suggested parietal TB that anti-mycobacterial treatment was commenced.

The macrosocopic appearance of the parietal pleural (Fig. 1) presented a rare discovery which, alongside histopathological findings (Figs2  3) aided in clinical diagnosis of MTB infection and confirmed the need for targeted therapy. Pleural TB is notoriously difficult to diagnose, not only owing to its wide spectrum of manifestations, but also due to the difficulty in obtaining optimal specimen from the pleural space [6,25]. When available, histopathological analysis can offer significant advantage if the AFBs are visualised in the sections, however, presence of granulomas alone is insufficient to distinguish MTB from other conditions such as scarcoidosis. A further disadvantage of histopathological diagnosis alone is that the species of acid-fast bacilli seen by Ziehl-Neelsen staining cannot be determined and no information is derived regarding antibiotic susceptibility [4,25]. These hurdles apply to other forms of extrapulmonary TB such as osteoarticular TB and are not unique to pleural TB [24]. Our case highlights the need to maintain mycobacterial culture facilities in microbiology laboratories despite the increasing use of rapid diagnostics for MTB. Our longitudinal data in this one illustrative case indicated that results of IGRAs may depend on the timing of the test along the spectrum of progression from latent to active disease. Conversion of a previously positive IGRA to negative IGRA result should prompt thorough investigation in cases with signs and symptoms suggestive of active TB.

## Abbreviations

AFB: acid fast bacilli
BAL: broncho-alveolar lavage
CFP-10: culture filtrate protein 10
CTPA: computed tomography pulmonary angiogram
ESAT-6: early secretory protein 6
IGRA: interferon-gamma release assay
LDH: lactate dehydrogenase
MGIT: mycobacterial growth indicator tube
MTB: *Mycobacterium tuberculosis*
PCR: polymerase chain reaction
PPD: purified protein derivative
QFG-GIT: quantiferon-gold in-tube
QFG-Plus: quantiferon-gold plus
TB: tuberculosis
WCC: white cell count
WHO: World Health Organisation
ZN: Ziehl Neelsen
